# Nasopharyngeal carcinoma derived exosomes regulate the proliferation and migration of nasopharyngeal carcinoma cells by mediating the miR-99a-5p BAZ2A axis

**DOI:** 10.1016/j.bjorl.2023.101343

**Published:** 2023-10-11

**Authors:** Xiao-jun Zhou, Hang-min Xu, Guo-sen Huang, Bao-rui Lin

**Affiliations:** a Integrated Hospital of Traditional Chinese Medicine of Southern Medical University, Department of Otolaryngology, Guangzhou, China; b Zhongshan Traditional Chinese Medicine Hospital Affiliated to Guangzhou University of Chinese Medicine, Zhongshan, China

**Keywords:** Nasopharyngeal carcinoma, Exosomes, MiR-99a-5p, BAZ2A, Proliferation, Migration

## Abstract

**Objectives:**

Nasopharyngeal Carcinoma (NPC) is a common malignant tumor of nasopharyngeal mucosal epithelium in clinical practice. Radiotherapy and chemotherapy are the main treatment methods at present, but the therapeutic effect is still unsatisfactory. Studies have shown that exosomes and microRNAs (miRNAs) play an important role in the development of cancer. Therefore, this study aimed to investigate the effects of NPC derived exosomes on NPC and their molecular mechanisms.

**Methods:**

Serum was collected from healthy subjects, Epstein–Barr Virus (EBV) infected patients and NPC patients (n = 9 group) and exosomes were extracted separately. High-throughput sequencing of exosomes was performed to screen differentially expressed miRNAs. The function of the screened miRNA was identified by treating NPC cells with exosomes. The target gene of miRNA was identified using the dual-luciferase assay. Real-Time quantitative Polymerase Chain Reaction (RT-qPCR) was used to determine the levels of miR-99a-5p and Bromodomain Adjacent Tozinc finger domain protein 2A (BAZ2A). Cell Counting Kit-8 assay, flow cytometry, and wound healing assay were utilized to detect cell viability, cell cycle and apoptosis, and migration ability. The protein levels were evaluated by Western blot.

**Results:**

MiR-99a-5p was identified as the most significant differentially expressed miRNA in exosomes (*p* < 0.05). The proliferation and migration of NPC cells were extremely facilitated by exosomes, accompanied by the suppressed apoptosis, upregulated BAZ2A, Monocyte Chemotactic Protein-1 (MCP1), and Vascular Endothelial Growth Factor A (VEGFA), and downregulation of Interleukin (IL)-1β and Nuclear Transcription Factor-κB (NF-κB) (*p* < 0.05). BAZ2A was a target gene of miR-99a-5p. Furthermore, the regulatory effect of exosomes on the proliferation, migration, and apoptosis was significantly abolished by overexpression of miR-99a-5p or downregulation of BAZ2A (*p* < 0.05).

**Conclusion:**

NPC derived exosomes facilitated the proliferation and migration of NPC through regulating the miR-99a-5p/BAZ2A axis.

## Highlights

• MiR-99a-5p was the most significant differentially expressed gene in exosomes.
• The proliferation and migration of NPC cells were extremely facilitated by exosomes.
• NPC derived exosomes facilitated the proliferation and migration of NPC.

## Introduction

Nasopharyngeal Carcinoma (NPC) is a malignant tumor located in the mucosal epithelium of the nasopharynx, which is a common head and neck tumor.^1^ Compared with common head and neck squamous cell carcinoma, NPC is sensitive to radiotherapy and chemotherapy with characteristic clinical manifestations and a better prognosis. With the development of radiotherapy technology and the optimization of chemotherapy regimens, the locoregional control rate of NPC has been significantly improved. However, 4-year Overall Survival (OS) remains low.^2^, ^3^ Radiation resistance are observed in approximately in 20% of NPC patients.^4^ Therefore, it is important to explore the novel mechanisms regulating the biological behavior of NPC cells.

Studies have shown that microRNAs (miRNAs) in exosomes bind to target genes related to various cellular pathways and participate in angiogenesis, cell transport, apoptosis, and protein cleavage.^5^, ^6^ Based on this, there are increasing studies on exosomal miRNAs in NPC. Jiang et al. showed that miR-197-3p in NPC cell-derived exosomes reduced the proliferation and migration potential of NPC cells, inhibited autophagy of NPC cells, and improved NPC radiosensitivity by targeting HSPA5 to regulate the AKT/mTOR pathway.^7^ Yang et al. found that miR-205-5p in NPC cell-derived exosomes enhanced EGFR/ERK signaling and MMP expression by targeting DSC2 and promoted angiogenesis and NPC metastasis.^8^ In addition, Li et al. showed that miR-106a-5p in NPC cell-derived exosomes activates AKT phosphorylation by targeting Aryl hydrocarbon Receptor Nuclear Translocator 2 (ARNT2), thereby promoting NPC cell proliferation, reducing apoptosis, and resistance to cisplatin.^9^ These studies suggest that exosomal miRNAs play a crucial role in regulating tumor development.

MiR-99a-5p, as a tumor suppressor gene, is a potential biomarker for predicting early squamous neck cell carcinoma.^10^ Studies have shown that miR-99a-5p regulates doxorubicin resistance through the COX-2/ABCG2 axis in triple-negative breast cancer.^11^ miR-99a-5p plays an anti-tumor role in tumor metastasis by targeting CDC25A/IL6 to hinder the Epithelial–Mesenchymal Transition (EMT) process in human cervical squamous cell carcinoma.^12^ Thus, miR-99a-5p is a candidate miRNA for inhibiting a variety of tumors. However, no study has yet reported the effect of miR-99a-5p in NPC.

The Bromodomain Adjacent Tozinc finger domain protein 2A (BAZ2A) plays an important role in chromatin remodeling and regulation of non-coding RNAs.^13^ Previous studies have shown that BAZ2A is an epigenetic regulator involved in ribosomal RNA transcription and is overexpressed in prostate cancer and is a marker for predicting prostate cancer recurrence.^14^ Recent studies have shown that TIP5 exerts oncogenic effects in Hepatocellular Carcinoma (HCC) by activating β-catenin/TCF7L2 signaling, suggesting that TIP5 may be a promising therapeutic target for HCC.^15^ LINC00885 exerts oncogenic effects in cervical cancer by adsorbing miR-3150b-3p through sponges and upregulating BAZ2A.^16^ These studies suggest that BAZ2A promotes tumor progression. However, the relationship between BAZ2A and NPC is unclear. In this study, binding sites of miR-99a-5p to BAZ2A were predicted by TargetScan database. However, these research gaps mentioned above are highly desirable to be explored experimentally.

Therefore, in this study, we screened differentially expressed miRNAs in serum by high-throughput sequencing and investigated the effects of serum-derived exosomes on NPC cell proliferation, migration and apoptosis. In addition, serum exosomes were added to NPC cells overexpressing miR-99a-5p to investigate the effect of serum exosomes on the miR-99a-5p/BAZ2A axis. Our study aimed to investigate the effect of serum-derived exosomes and miR-99a-5p/BAZ2A axis on NPC, thus providing more scientific basis for the pathogenesis and treatment of NPC.

## Methods

### The extraction and identification of exosomes

The serum was moved into a new centrifuge tube and centrifuged at 2000×*g* for 30 min at 4 °C. The supernatant was carefully transferred to a new centrifuge tube and centrifuged at 10,000×*g* and 4 °C for 45 min to remove larger vesicles. The supernatant was removed and filtered through a 0.45 μm filter membrane (HAWG04700, Sigma-Aldrich.cn) to collect the filtrate, which was transferred to a new centrifuge tube and centrifuged at 100,000×*g* for 70 min at 4 °C with a superspeed rotor selected. After removal of the supernatant and resuspension with 10 mL of precooled Phosphate Buffered Saline (PBS), the rotor was selected and ultra-centrifugated at 100,000×*g* at 4 °C for 70 min. The precipitates were collected as exosomes, which were resuspended in 800 μL of precooled PBS for subsequent transmission electron microscopy (Olympus TM3030Plus, Japan), particle size identification, and miRNA high-throughput sequencing. This study has been approved by the Ethics Committee (Zhongshan Traditional Chinese Medicine Hospital Ethics Committee), and all patients have signed informed consent.

### High-throughput sequencing of miRNA

Exosomes were extracted from three types of serum: serum from 9 healthy subjects (NC’exo), serum from 9 Epstein–Barr Virus (EBV) infected patients (EBV’exo), and serum from 9 NPC patients (NPC’exo). Exosomes obtained by ultracentrifugation were added to the lysate to extract RNA, and a small RNA sequencing library was constructed. High-throughput sequencing of miRNA was performed on the Illumina platform. The differentially expressed miRNAs were screened and verified by quantitative Polymerase Chain Reaction (qPCR).

### Real-time quantitative PCR (RT-qPCR)

RNAs were extracted from cells using the Trizol Reagent (CW0580S, CWBIO, China), followed by obtaining the total miRNA using the miRNA extraction kit (CW0627S, CWBIO, China) and the total mRNA using the mRNA extraction kit (CWBIO, China). Total miRNA and mRNA were transcribed into micDNA and cDNA using the miRNA cDNA Synthesis Kit (CW2141S, CWBIO, China) and the HiScript II Q RT SuperMix for qPCR (+gDNA wiper) (R223-01, Vazyme, China), respectively. The PCR reaction was conducted with the miRNA Universal SYBR qPCR Master Mix (MQ101-02, Vazyme, China) and ChamQ Universal SYBR qPCR Master Mix Q711-02, Vazyme, China). Real-time fluorescent quantitative PCR instrument Gene amplification instrument system (ABI 7500, ThermoFisher Scientific) was used for PCR detection. The gene levels were checked utilizing the 2^−ΔΔCt^ method. Sequences of primers were shown in Table 1. U6 and β-actin were utilized as the internal reference for miRNA and mRNA, respectively.

**Table 1:** Sequences of primers used in the RT-PCR assay.

Gene	Forward primer (5′ → 3′)	Reverse primer (5′ → 3′)
U6	CTCGCTTCGGCAGCACA	AACGCTTCACGAATTTGCGT
MiR-99a-5p	GCGAACCCGTAGATCCGAT	AGTGCAGGGTCCGAGGTATT
BAZ2A	GCCTCGGTACTCGGAAGAAG	CATCTCCATCAGGATAATCTCGCA
β-Actin	TGGCACCCAGCACAATGAA	CTAAGTCATAGTCCGCCTAGAAGCA

### Western blotting assay

The BCA kit (Cwbio, Jiangsu, China) was utilized to quantify the protein isolated from cells, followed by being separated with the 12% SDS-PAGE. The separated protein was transferred from the gel to the Polyvinylidene Difluoride Membrane (PVDF) membrane, which was further introduced with 5% skim milk. Then, the membrane was introduced with the primary antibody against BAZ2A (1:1000, DF3858, Affinity, USA), Interleukin (IL)-1β (1:1000, AF5103, Affinity, USA), Monocyte Chemotactic Protein 1 (MCP1, 1:1000, DF7577, Affinity, USA), Nuclear transcription Factor-κB (NF-κB, 1:1000, 66535-1-Ig, Proteintech, USA), Vascular Endothelial Growth Factor A (VEGFA, 1:1000, AF5131, Affinity, USA), and GAPDH (1:2000, TA-08, ZSBIO, China). The second antibody (1:2000, ZB-2301, ZSBIO, China) was subsequently added to be incubated for 90 min. Finally, ECL reagent was added to expose the bands, which were further quantified with the Image J software.

### CCK-8 assay

Cells were implanted in 96-well plates for 24 h, followed by adding with 10 μL CCK8 solution. After incubating for 2 h, the OD value was detected using the microplate reader (WD-2012B, LIUYI, China).

### Wound healing assay

When the cell density reached more than 90%, 200 μL of the gun tip was used to scratch in each well, followed by discarding the medium and replaced with DMEM incomplete medium. Then the scratch in each well was photographed. Cells were put into the incubator, and the scratch of each well was photographed again after 24 h. According to the scratch condition, the 24 h scratch data and the 0 h scratch data were determined. The corresponding scratch width and migration rate was calculated.

### The detection of apoptosis using the flow cytometry

1 × 10^6^ cells were collected and washed by PBS buffer, which were then re-suspended with 300 μL pre-cold 1× Annexin V-FITC binding buffer. Then, cells were introduced with 5 μL Annexin V-FITC reagent and 10 μL PI reagent, followed by 10 min incubation in the dark at room temperature. Lastly, cells were loaded onto the flow cytometry (NovoCyte 2060R, ACEA Biosciences, China) for the analysis of apoptosis.

### The detection of cell cycle using the flow cytometry

The medium in the 6-well plate was discarded, and each well was rinsed twice with 1 mL PBS. Then 200 μL EDTA containing trypsin digestion solution was added and digested in 37 °C incubator. The cell suspension was centrifuged at 3000 rpm for 3 min, and the supernatant was discarded. 1 mL PBS was added to each tube, followed by centrifuged at 3000 rpm for 3 min. 1 mL of absolute ethanol precooled at 4 °C was added to each tube and placed in a 4 °C refrigerator for more than 2 h. Samples were centrifuged at 3000 rpm for 3 min and the supernatant was discarded. 1 mL of PBS was added, followed by centrifuged at 3000 rpm for 3 min and discarding the supernatant. 500 μL DNA staining solution was added to be incubated in the dark for 1 h, followed by loaded onto the flow cytometry (NovoCyte 2060R, ACEA Biosciences, China) for the analysis of cell cycle.

### Double luciferase assay

The full-length 3′-UTR fragment of the BAZ2A gene was amplified using the BEC genome as a template, and the PCR product was digested, followed by linking the amplified product to the pMIR plasmid. After transforming the amplified product into DH5α cells, positive clones were screened and sequenced for correction. The correct sequencing plasmids were used for transfection and named pMIR-BAZ2A-WT and pMIR-BAZ2A-MUT. Subsequently, pMIR-BAZ2A-WT or pMIR-BAZ2A-MUT was transfected into HEK293T cells, along with the miR-99a-5p mimic or mimic NC, using Lipofectamine 2000. After 2-day incubation, relative luciferase activity was measured according to the instructions of the Dual-Glo Luciferase Assay System Kit.

### The grouping for the verification of miR-99a-5p function in NPC

Four groups were divided to verify the miR-99a-5p function in NPC: Control, NPC’exo, NPC’exo+ mimic, and NPC’exo+ mimic NC. In the control group, NPC cells were treated with blank medium. In the NPC’exo group, NPC cells were treated with 20 μg/mL NPC’exo, while in the NPC’exo+ mimic group, NPC cells were treated with 20 μg/mL NPC’exo combined with 50 nM miR-99a-5p mimic. In the NPC’exo+ mimic NC group, NPC cells were treated with 20 μg/mL NPC’exo combined with 50 nM mimic NC.

### Statistical analysis

Mean ± Standard Deviation (SD) was utilized to present data, which was analyzed using the one-way ANOVA method with the software of GraphPad prism software 6.0; *p* < 0.05 was considered to be a statistically significant difference.

## Results

### The identification of extracted exosomes

Exosomes are derived from multivesicular bodies in cells, which are membrane vesicles with a diameter of about 30–150 nm secreted by living cells. The density of exosomes is 1.13–1.19 g/mL with a typical “cup and plate” shape.

**Figure 1:**
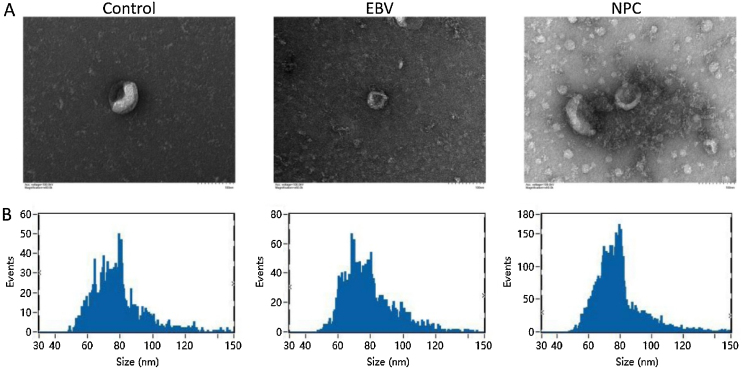
The extracted exosomes were identified. (A) The morphology of exosomes was imaged using the transmission electron microscopy. (B) The particle size of exosomes was detected by the Nanoparticle Tracking Analysis (NTA).

As shown in Fig. 1A‒B, exosomes extracted from each group showed a typical “cup and plate” morphology, and the particle size was mainly distributed between 50 and 120 nm, which was consistent with the basic characteristics of exosomes.

### High-throughput sequencing of miRNAs and identification

**Figure 2:**
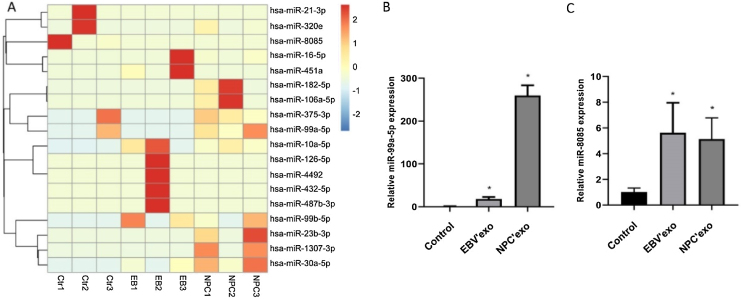
Mir-99a-5p and miR-8085 were significantly highly expressed in EBV’exo and NPC’exo. (A) Screening of differentially expressed miRNAs in exosomes from healthy subjects’ sera (NC’exo), EBV-infected patients’ sera (EBV’exo) and NPC patients’ sera (NPC’exo) by high-throughput sequencing of miRNAs. (B‒C) RT-qPCR was used to detect miR-99a-5p (B) and miR-8085 (C) expression levels in NC’exo, EBV’exo and NPC’exo (*p < 0.05 vs. control).

MiR-99b-5p and miR-8085 were selected as candidate genes by high-throughput sequencing analysis Fig. 2A, which were subsequently validated by qPCR. As shown in Fig. 2B‒C, compared to control, miR-99a-5p and miR-8085 were significantly upregulated in EBV’exo (miR-99a-5p expression, 18.02 ± 4.82; miR-8085 expression, 5.63 ± 2.33; n = 3), and NPC’exo (miR-99a-5p expression, 259.72 ± 23.80; miR-8085 expression, 5.14 ± 1.64; n = 3), in which miR-99a-5p showed the most significant differential expression. Therefore, miR-99a-5p was selected for subsequent assays.

### The impact of different exosomes on the biofunction of NPC cells

**Figure 3:**
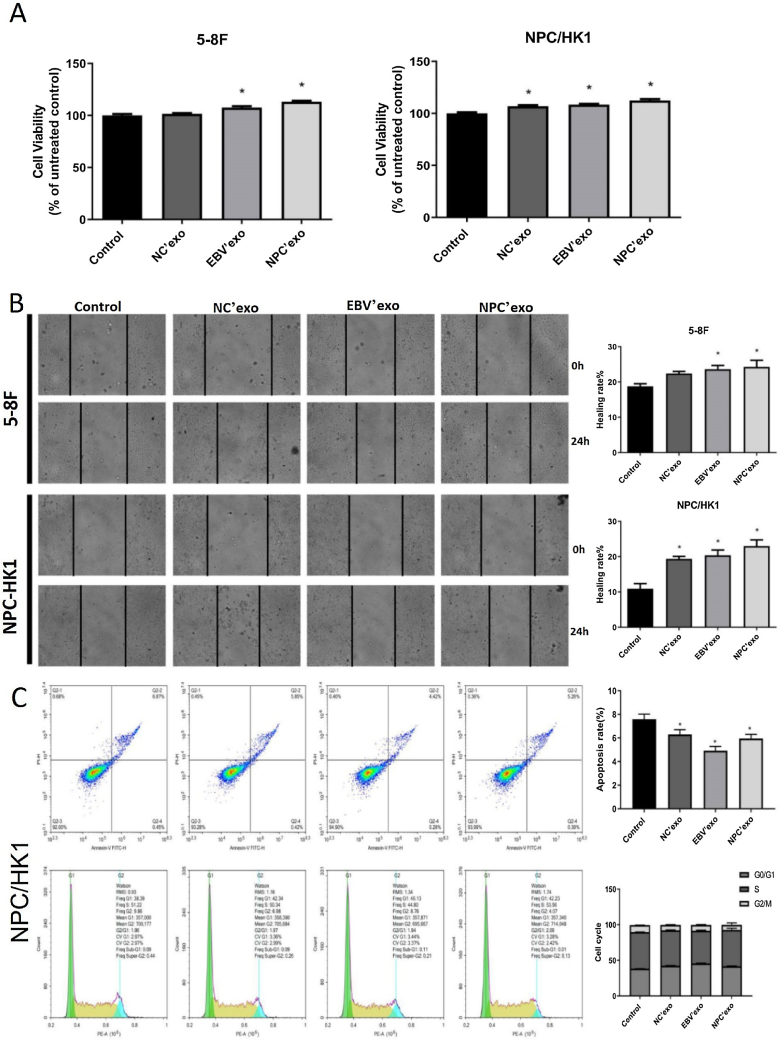
The impact of different exosomes on the biofunction of NPC cells. (A) The cell viability was detected by the CCK-8 assay. (B) The migration ability of NPC cells was evaluated by the wound healing assay. (C) The apoptosis and cell cycle were detected by the flow cytometry (*p < 0.05 vs. control).

As shown in Fig. 3A‒B, in both 5‒8F and NPC/HK1 cells, compared to control, the cell viability and migration rate were extremely elevated by the introduction of EBV’exo and NPC’exo. Furthermore, higher cell viability (EBV’exo group 107.59 ± 2.87 and NPC’exo group 113.21 ± 2.08 in 5‒8F cell, compared to the control group 100 ± 3.1; EBV’exo group 108.41 ± 1.69 and NPC’exo group 112.41 ± 3.12 in NPC/HK1 cell, compared to the control group 100 ± 3.12; n = 5) and migration rate (EBV’exo group 23.62 ± 2.42 and NPC’exo group 24.29 ± 4.21 in 5‒8F cell, compared to the control group 18.78 ± 1.64; EBV’exo group 20.39 ± 3.34 and NPC’exo group 23.00 ± 3.94 in NPC/HK1 cell, compared to the control group 10.92 ± 3.21; n = 3) were observed in the NPC’exo compared to the EBV’exo group. As shown in Fig. 3C, in NPC/HK1 cells, compared to control, the apoptotic rate was greatly reduced by the introduction of EBV’exo (4.92 ± 0.36; n = 3) and NPC’exo (5.96 ± 0.34; n = 3). Moreover, no significant difference was observed among different groups regarding the cell cycle.

### The impact of different exosomes on the protein level of BAZ2A, IL-1β, MCP1, NF-κB, and VEGFA in NPC cells

**Figure 4:**
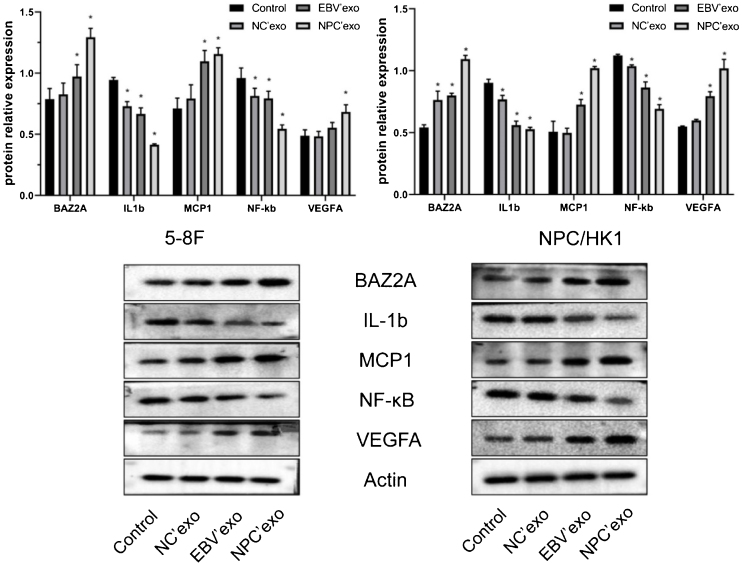
The expression level of BAZ2A, IL-1β, MCP1, NF-κB, and VEGFA in NPC cells treated by different exosomes was detected by the Western blotting assay (*p < 0.05 vs. control).

As shown in Fig. 4, compared with the control group, the expression levels of BAZ2A (EBV’exo group 0.97 ± 0.1 and NPC’exo group 1.29 ± 0.07 in 5‒8F cell; EBV’exo group 0.80 ± 0.02 and NPC’exo group 1.09 ± 0.03 in NPC/HK1 cell; n = 3), MCP1 (EBV’exo group 1.10 ± 0.09 and NPC’exo group 1.16 ± 0.05 in 5‒8F cell; EBV’exo group 0.73 ± 0.04 and NPC’exo group 1.02 ± 0.01 in NPC/HK1 cell; n = 3) and VEGFA (EBV’exo group 0.55 ± 0.04 and NPC’exo group 0.68 ± 0.06 in 5‒8F cell; EBV’exo group 0.79 ± 0.04 and NPC’exo group 1.02 ± 0.07 in NPC/HK1 cell; n = 3) protein were significantly up-regulated, and the expression levels of IL-1β (EBV’exo group 0.67 ± 0.05 and NPC’exo group 0.41 ± 0.01 in 5‒8F cell; EBV’exo group 0.56 ± 0.03 and NPC’exo group 0.53 ± 0.02 in NPC/HK1 cell; n = 3) and NF-κB (EBV’exo group 0.79 ± 0.06 and NPC’exo group 0.55 ± 0.03 in 5–8F cell; EBV’exo group 0.86 ± 0.05 and NPC’exo group 0.69 ± 0.03 in NPC/HK1 cell; n = 3) protein were significantly down-regulated in NPC cells treated with EBV’exo and NPC’exo. In addition, the expression levels of BAZ2A, MCP1 and VEGFA protein in NPC’exo group were significantly higher than those in NC’exo group, while the expression levels of IL-1β and NF-κB protein were significantly lower than those in NC’exo group.

### The binding between miR-99a-5p and BAZ2A was identified by the dual-luciferase assay

**Figure 5:**
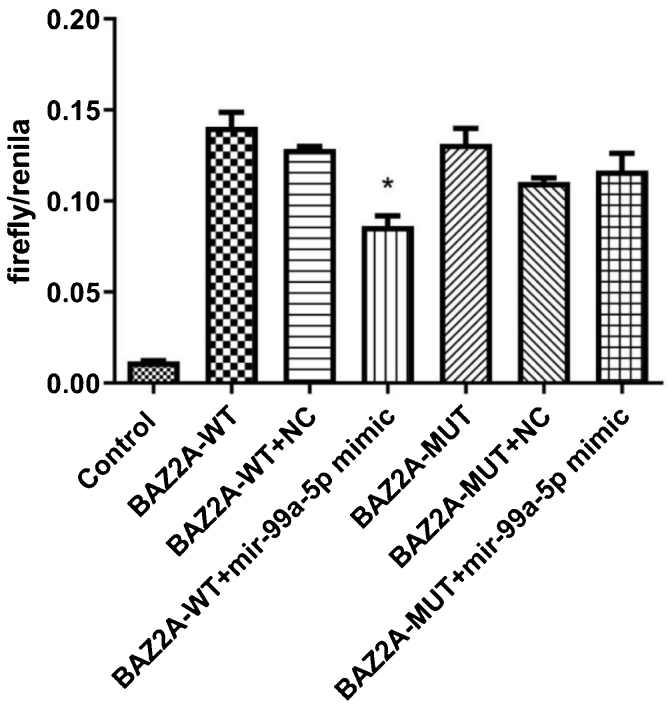
The binding between miR-99a-5p and BAZ2A was identified by the dual-luciferase assay (*p < 0.05 vs. BAZ2A-WT).

As shown in Fig. 5, compared to the BAZ2A-WT + NC group (590.00 ± 41.59; n = 9), dramatically declined fluorescence intensity was observed in the BAZ2A-WT + miR-99a-5p mimic group (398.56 ± 75.50; n = 9). However, no significant difference on the fluorescence intensity was observed between the BAZ2A-WUT + NC group (520.11 ± 38.61; n = 9) and BAZ2A-WUT miR-99a-5p mimic groups (543.56 ± 64.33; n = 9). These data suggested that miR-99a-5p specifically targeted BAZ2A.

### The identification of miR-99a-5p overexpression in NPC cells

**Figure 6:**
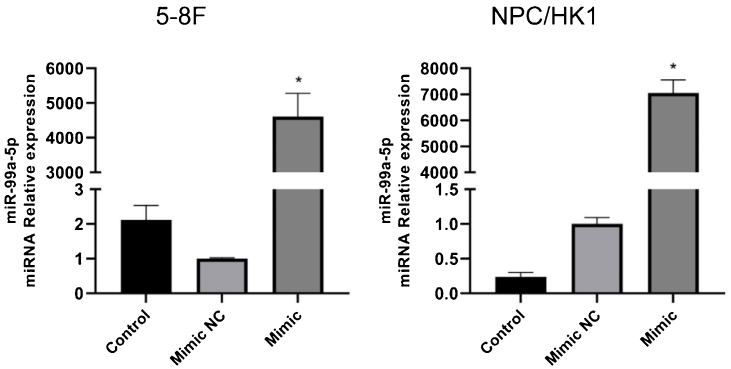
The transfection efficacy of miR-99a-5p in NPC cells was identified by the RT-PCR assay (*p < 0.05 vs. mimic NC).

To overexpress miR-99a-5p in NPC cells, cells were transfected with miR-99a-5p mimic, with mimic NC as a negative control. As shown in Fig. 6, in both 5‒8F and NPC/HK1 cells, compared to mimic NC (1.00 ± 0.03 in 5‒8F cell, 1.00 ± 0.09 in NPC/HK1 cell; n = 3), the level of miR-99a-5p (4610.04 ± 669.21 in 5‒8F cell, 7054.10 ± 501.77 in NPC/HK1 cell; n = 3) was extremely elevated by the transfection of miR-99a-5p mimic, suggesting a successful overexpression of miR-99a-5p in NPC cells.

### The verification of miR-99a-5p function in NPC cells

**Figure 7:**
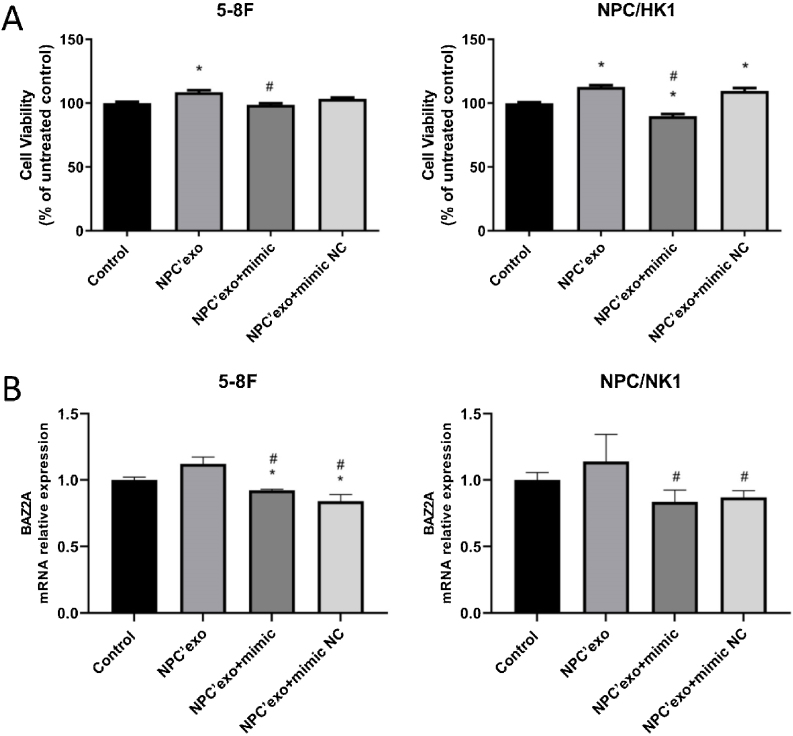
The verification of miR-99a-5p function in NPC cells. (A) The cell viability was detected by the CCK-8 assay. (B) The level of BAZ2A in NPC cells was measured by the RT-PCR assay (*p < 0.05 vs. control, #*p* < 0.05 vs. NPC’exo).

As shown in Fig. 7A, in both 5–8F and NPC/HK1 cells, compared to control (100.00 ± 2.51 in 5‒8F cell, 100.00 ± 1.43 in NPC/HK1 cell; n = 5), the cell viability was extremely elevated in the NPC’exo (108.54 ± 3.59 in 5‒8F cell, 112.69 ± 3.08 in NPC/HK1 cell; n = 5), which was greatly repressed in the NPC’exo+ mimic group (98.66 ± 2.82 in 5‒8F cell, 89.83 ± 3.97 in NPC/HK1 cell; n = 5), suggesting that the effect of miR-99a-5p on the proliferation of NPC cells was partly abolished by miR-99a-5p.

As shown in Fig. 7B, in both 5–8F and NPC/HK1 cells, compared to NPC’exo (1.12 ± 0.05 in 5‒8F cell, 1.14 ± 0.21 in NPC/HK1 cell; n = 3), the BAZ2A level was dramatically reduced in the NPC’exo+ mimic group (0.92 ± 0.01 in 5‒8F cell, 0.84 ± 0.09 in NPC/HK1 cell; n = 3), suggesting that BAZ2A was downregulated by the overexpression of miR-99a-5p.

## Discussion

NPC is the most common malignant tumor of the head and neck in China, which has a unique geographical incidence pattern. NPC is highly prevalent in southern China, with an annual incidence of about 30 cases per 100,000 people. The incidence of NPC is related to a variety of risk factors, including EBV infection, genetic susceptibility, and environmental factors.^1^

The secretion of exosomes is commonly observed in tumor tissues and cells, which are involved in angiogenesis, tumor microenvironment remodeling, and regulation of cancer metastasis and drug resistance.^17^ We extracted exosomes from serum in healthy subjects, EBV infected patients, and NPC patients, respectively. The typical “plates” morphology and 50‒120 nm particle size indicated that exosomes were successfully extracted.^18^ By high-throughput sequencing, miR-99b-5p and miR-8085 were screened out and miR-99b-5p showed a more significant difference. As a tumor suppressor, miR-99a-5p is reported to inhibit cell proliferation and migration, which is downregulated in tumor tissues.^19^ In the present study, miR-99a-5p was highly expressed in peripheral blood, which may be due to the regulation of miRNAs in peripheral blood by complex feedback mechanisms^20^ or the different or even opposite roles of miRNAs in different tissues or cells. For example, miRNA-221 and 222 show anti-proliferation and pro-apoptosis effects in endothelial cells.^21^ However, opposite effects in smooth muscle cells are observed.^22^ Exosomes are claimed to affect the biological function of cells by replicating the growth microenvironment of parent cells.^23^ Exosomes from glioblastoma is found to promote the proliferation and drug resistance of glioblastoma cells in vitro.^24^ In the present study, the cell proliferation and migration ability of NPC cells were markedly facilitated by EBV’exo and NPC’exo, accompanied by a declined apoptotic rate, indicating that NPC exosomes promoted the proliferation and migration, and inhibited cell apoptosis by creating a similar tumor microenvironment. However, no significant difference in cell cycle among groups was observed, which might be driven by the interaction between multiple kinases and related cyclins.

Western blotting was used to detect the expression of BAZ2A, MCP1, and VEGFA in 5‒8F and NPC/HK1 cells treated by three types of exosomes, which was found dramatically elevated by the treatment of EBV’exo and NPC’exo. BAZ2A is highly expressed in metastatic tumors and promotes tumor cell proliferation and migration.^14^ MCP1 is a cell chemotactic factor related to cell migration, which is also reported to facilitate the migration of smooth muscle cells.^25^ When the level of MCP1 is increased, enhanced infiltration of Tumor-Associated Macrophages (TAM) will be triggered, thereby contributing to the tumor growth.^26^ Vascular Endothelial Growth Factor A (VEGFA) is highly expressed in tumor tissues and induces angiogenesis.^27^ Overexpression of VEGFA is found to promote the survival and capillar-like tube formation of paranasal Sinus Squamous Cell Carcinoma (SNSCC) cells, and inhibit the apoptosis.^28^ Results obtained in the present study identified that BAZ2A, MCP1 and VEGFA played a key role in the proliferation and migration of NPC cells. Furthermore, BAZ2A was identified as the target of miR-99a-5p in NPC cells, suggesting that miR-99a-5p might exert regulatory function in NPC cells by mediating the expression of BAZ2A.

It has been reported that calcitriol inhibits the proliferation of triple-negative breast cancer cells by stimulating the expression of IL-1β and TNF-α.^29^ NF-kB is considered as an oncogene, which has been targeted for the cancer treatment. However, recent evidence suggests that NF-κB may also functions as a tumor suppressor, inhibition of which instead increases the proliferative viability of ovarian cancer cells.^30^ In the present study, the low expression of IL-1β and NF-κB in NPC cells implied that inhibition on IL-1β and NF-κB might result in the facilitated proliferation and migration.

Although our study suggests that NPC derived exosomes can promote NPC progression and may be through regulation of the miR-99a-5p/BAZ2A axis. However, many unresolved questions remain in our study. First, we did not further investigate the effect of NPC derived exosomes on proliferation, migration and invasion of NPC cells overexpressing miR-99a-5p, as well as further validate our results by reversion assays. In addition, we did not investigate the effect of NPC derived exosomes on NPC progression by regulating the miR-99a-5p/BAZ2A axis in an animal model. Finally, the number of samples in each group sequenced in this study was small, and more clinical samples are needed to further identify miR-99a-5p as a potential target for NPC in the future. Therefore, the shortcomings in these studies will continue to be explored in depth later, thus providing more insights into the role of miR-99a-5p and BAZ2A in NPC.

## Conclusions

In short, In the present study, NPC derived exosomes can significantly promote NPC cell proliferation and migration and inhibit apoptosis, however NPC cells overexpressing miR-99a-5p can partially eliminate these effects of exosomes, and the miR-99a-5p/BAZ2A axis may be the regulatory mechanism.

## Availability of data and materials

Data involved in the present work are available from corresponding author upon request.

## Funding

This work was partially supported by 10.13039/501100001809National Natural Science Foundation of China (81373698); Project of Guangdong Administration of Traditional Chinese Medicine (20201420, 20221427).

## Ethical approval

This study was approved by the Ethics Committee of Integrated Hospital of Traditional Chinese Medicine.

## Conflicts of interest

The authors declare no conflicts of interest.
